# Cytokine/chemokine levels in the CSF and serum of anti-NMDAR encephalitis: A systematic review and meta-analysis

**DOI:** 10.3389/fimmu.2022.1064007

**Published:** 2023-01-23

**Authors:** Yushan Ma, Jierui Wang, Shuo Guo, Zirui Meng, Yan Ren, Yi Xie, Minjin Wang

**Affiliations:** ^1^ Department of Laboratory Medicine, West China Hospital of Sichuan University, Chengdu, China; ^2^ Department of Laboratory Medicine, The Third Hospital of Mianyang, Sichuan Mental Health Center, Mianyang, China; ^3^ Department of Neurology, West China Hospital of Sichuan University, Chengdu, China

**Keywords:** cytokine, chemokine, T cell, NMDAR encephalitis, meta-analysis

## Abstract

**Objectives:**

To summarize the cytokine/chemokine levels of anti-N-methyl-Daspartate receptor encephalitis (NMDAR-E) and explore the potential role of these molecules and immune cells in the pathogenic mechanism.

**Methods:**

The PubMed, Cochrane Library, Embase, and Web of Science databases were searched for various articles that assessed the concentrations of cytokines/chemokines in the unstimulated cerebrospinal fluid (CSF) or serum of patients with NMDAR-E in this systematic review and meta-analysis. The standardized mean difference (SMD) and 95% confidence interval (CI) were calculated by Stata17.0.

**Results:**

A total of 19 articles were included in the systematic review from 260 candidate papers, and cytokine/chemokine levels reported in the CSF/serum were examined in each article. This meta-analysis included 17 eligible studies comprising 579 patients with NMDAR-E, 367 patients with noninflammatory neurological disorders, and 42 healthy controls from China, Spain, South Korea, Australia, Czechia, and Sweden. The results indicated that the levels of different cytokines interleukin (IL)-6, tumor necrosis factor (TNF)-α, IL-10, IL-13, IL-1β, IL-12, and IL-17 and chemokine C-X-C motif ligand (CXCL)10 in the CSF were significantly higher in NMDAR-E patients with a large effect size. In addition, B cell activating factor (BAFF), CXCL13, and interferon (IFN)-γ levels in the CSF were higher in NMDAR-E patients with a middle effect size. In contrast, levels of IL-2 and IL-4 in the CSF and CXCL13 and BAFF in the serum did not show a significant difference between cases and controls.

**Conclusions:**

These analyses showed that the central immune response in NMDAR-E is a process that involves multiple immune cell interactions mediated by cytokines/chemokines, and T cells play an important role in the pathogenesis of immunity.

**Systematic review registration:**

https://www.crd.york.ac.uk/PROSPERO/, identifier (CRD42022342485).

## 1 Introduction

Anti-N-methyl-D-aspartate receptor encephalitis (NMDAR-E) is one of the most common types of autoimmune neurological disorders, which affects young women more often than it does men (1–3). The clinical manifestations are often variable, including seizures, psychiatric behavior changes, and rapid cognitive decline (2, 4, 5). The majority of NMDAR-E patients have intrathecal antibody synthesis, which can cause a significant decrease in the cell surface and synaptic NMDAR by specific immunoglobulin G (IgG) antibodies against the GluN1 subunit on the neuronal membrane (6). Clinically, it is generally assumed that the progressive deficiency of the receptor function with increased antibody titer can reflect the severity of the disease and/or may be the primary cause of aggravation (7). However, since ongoing intrathecal antibody production does not always signify active encephalitis, this conclusion has certain limitations (8, 9).

Recently, few studies have reported that the key immune pathogenesis of NMDAR-E is often accompanied by immune dysregulation, which is inflammatory in nature. It is usually characterized by massive infiltration of T cells, B cells, and other immune cells into the central nervous system (CNS) (10, 11), triggering substantial neuron damage, demyelination, or neurodegeneration (12, 13). However, although the role of B cells in NMDAR-E has already been demonstrated (7, 14, 15), whether T cells can effectively react against NMDAR and their specific role in disease development remain unclear (16). Moreover, the mechanisms through which they can access the CNS compartment are unclear. Additionally, because T cells are predominantly found in the cerebrospinal fluid (CSF) of affected people while B cells are scarce (17), some prior studies have suggested that NMDAR-E onset could be potentially triggered by the activation of T cells in the periphery and CNS (13). Indeed, current pathological examination and postmortem reports further support the observation that the different effector T helper (Th) cells, such as Th1 and Th17 cells, could extensively be infiltrated in the tissues (11, 13, 14, 18). In particular, previous studies have demonstrated that Th cells can significantly enhance antibody-secreting cell (ASC)-mediated antibody responses, as mice were actively immunized with conformationally stabilized NMDAR protein and both ASCs and CD4+ T cells demonstrated extensive infiltration into the hippocampus (19). This finding indicated that the immune effects of one or several T cells may be important for regulating disease etiology and progression in NMDAR-E patients.

Cytokines/chemokines are low-molecular-weight polypeptides with biological activity mainly derived from the diverse antigen-presenting cells and mononuclear phagocytic cells (20, 21). As important immunoregulatory factors, cytokines/chemokines play a key role in the activation, differentiation, and migration of immune cells (22). In addition, various studies have revealed that dysregulation of the cytokine system plays a key role in neuroinflammatory disorders, and the alteration of cytokine levels has been increasingly linked to NMDAR-E. Interestingly, multiple studies including our own have suggested that CSF cytokine/chemokine signatures, such as elevated levels of interleukin (IL)-17, IL-6, or CXCL10, also suggest the possible involvement of T cells in the etiology of NMDAR-E (23–25). However, there is no broad consensus on these conclusions among the scientific community. This systematic review and meta-analysis focused on analyzing the previous studies related to CSF and serum cytokines and chemokines of NMDAR-E. Our research not only concentrated on the broad range of chemokines and cytokines associated with T-cell activities and examined how their levels were altered during the various clinical courses of NMDAR-E. More importantly, the detailed investigation of these molecules is expected to advance our comprehension of the disease’s immune mechanisms.

## 2 Methods

### 2.1 Search strategy

This systematic review and meta-analysis were conducted according to the preferred reporting items for systematic reviews and meta-analyses (PRISMA) criteria recommended for reporting items for systematic reviews and meta-analyses. This meta-analysis has been registered on the PROSPERO (No. CRD42022342485). We searched the PubMed, Web of Science, Embase, and Cochrane Library databases to identify articles published up to 25 June 2022 using a combination of the following keywords: (Anti NMDA Receptor Encephalitis OR anti-N-methyl-d-aspartate receptor encephalitis OR Anti-NMDAR Encephalitis OR N-methyl-d-aspartate antibody encephalitis OR NMDA receptor encephalitis OR anti-N-methyl-d-aspartate receptor antibody encephalitis) AND (cytokine OR interleukin OR interferon OR tumor necrosis factor-alpha OR transforming growth factor OR chemokine). The detailed search strategy used for each database has been described in the *Supplementary Methods*.

### 2.2 Selection criteria

The inclusion criteria were as follows: 1) the types of studies including cross-sectional, case–control, and cohort; 2) the level of anti-NMDAR antibody was found to be positive in the serum or CSF; 3) other autoantibody tests of the specimen were negative; 4) assessed the concentrations of the different cytokines/chemokines in the unstimulated serum or CSF with noninflammatory neurological disorder control or healthy control group.

The exclusion criteria were as follows: 1) reviews, case reports, experiments on animals or cells, conference papers, and editorial materials; 2) incomplete full text or nonconforming data; 3) different studies that mainly used reduplicated data.

### 2.3 Study selection and data extraction

YM and JW, two reviewers, each separately searched the articles, imported them into Endnote X9, and managed the search records. Thereafter, by using the Newcastle–Ottawa Scale (NOS), we also evaluated the quality of the papers that were included. The initial author’s name, research period, location, the source of the control group, sample size, gender, mean/median age, and quality scores were then collected as key data from a few selected studies.

The major results were the variation in the mean levels of inflammatory cytokines and chemokines between each case group and control group. The information about the number of participants, mean levels, and the standard deviation (SD) for cytokines/chemokines of NMDAR-E and control was obtained to calculate the mean difference. If the mean or SD was not stated directly in the articles, we could estimate them from the sample size and the alternative measures, including median, range, and 95% confidence interval (CI) or interquartile range, utilizing Wan and Luo’s online data converter (26, 27). If these measures were not available, the data were extracted from the figures and graphs by using an analogy to the approach as described in Saghazadeh and Rezaei (28).

### 2.4 Statistical analysis

In our study, Stata17.0 was used for all data analyses. Meta-analyses were performed when cytokine/chemokine data were extracted from at least three studies. The standardized mean difference (SMD) calculated by Hedges’ g was taken to analyze the effect with measurement scale differences across the studies, and its 95% CI was used as the result of the pooled analysis. Then, by using the SMD cutoff points of 0.2, 0.5, and 0.8, effect sizes were also broadly classified as small, medium, and large. We used the random-effects model (DerSimonian–Lard) for each meta-analysis to obtain a satisfactory estimator of heterogeneity variance for the continuous results.

Q statistic test was used to determine whether heterogeneity was presented across the studies of each factor, and tau-squared (t^2^) was a quantitative measurement of heterogeneity, while the I-squared (I^2^) was the heterogeneity across the studies from the total heterogeneity. The significance level was considered at *P* < 0.1.

To ensure that the results were robust and reliable, a sensitivity analysis was performed to determine whether the significance of the calculated effect size was sensitive to the effect when every single study was omitted. Thus, to assess the potential publication bias, if there were more than 10 research studies published, we will use a funnel plot and Egger test, where *P* < 0.05 was deemed statistically significant.

## 3 Results

### 3.1 Literature search and selection

**Figure 1:**
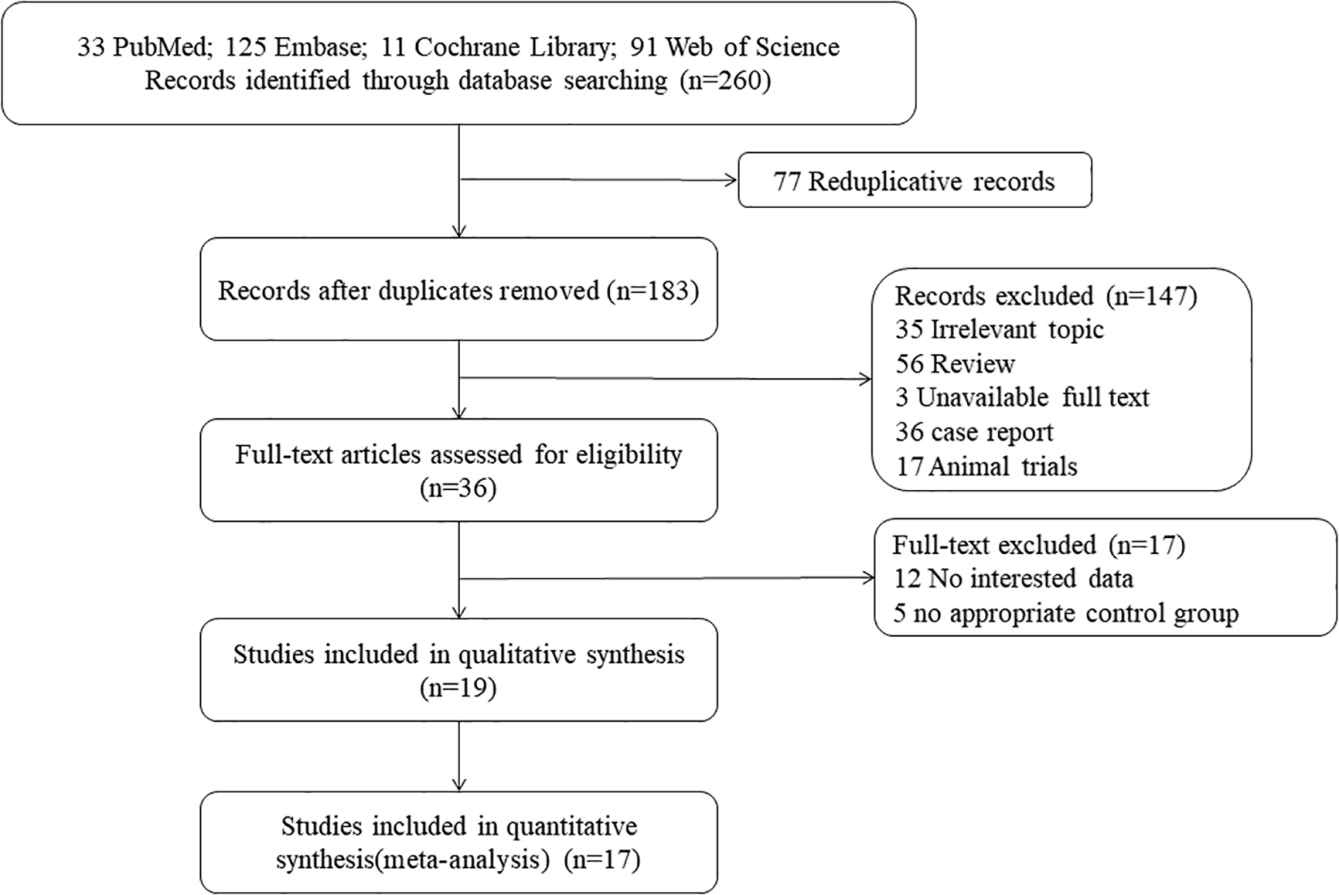
The procedures involved in study screening.

A total of 260 candidate articles were initially identified, and 36 studies were considered for further review after in-depth analyses of all of the titles and abstracts. It was found that among these studies, 12 had no interesting data and five had no appropriate control group; 17 studies meeting the inclusion requirements were included in the meta-analysis, and 19 studies were in the quantitative synthesis. The detailed procedure of the selection has been illustrated in **Figure 1**.

### 3.2 Characteristics of the included studies

Overall, 19 studies met the inclusion and exclusion criteria, and we stipulated that if a certain cytokine or chemokine was measured in more than three studies, then it can be included in the meta-analysis (23, 24, 29–45). cluster of differentiation 146 (CD146) and high- mobility group box protein 1 (HMGB1) concentrations of NMDAR-E were only found in a single study each; these two studies were excluded from the meta-analysis as a result of insufficient data (33, 43). In addition, there were a few studies about the serum cytokine levels in NMDAR-E patients, and in some studies, which only measured cytokine levels in the whole autoimmune encephalitis, data about NMDAR-E could not be extracted from the overall data and were also not available from the authors (46, 47). For these reasons, 37 cytokines/chemokines of the CSF and 21 cytokines/chemokines of the serum were not analyzed for having less than three studies, including a proliferation-inducing ligand (APRIL), cluster of differentiation 40 ligand (CD40L), C-C motif ligand(CCL)2 (CCL2), IL-8, granulocyte-macrophage colony-stimulating factor (GM-CSF), CCL17, CCL19, HMGB1, chitinase-3-like protein 1 (YKL-40), NOD-like receptor thermal protein domain associated protein 3 (NLRP3), CD146, osteopontin, etc. (23, 24, 30, 31, 33–35, 38, 40, 42, 43).

Meta-analyses of the different cytokines/chemokines in 13 CSF and two serum studies were performed. The two cytokines found in the CSF with the most studies were IL-17 and IL-6. A total of 579 NMDAR-E patients and 409 controls from China, Spain, South Korea, Australia, Czechia, and Sweden were included. The characteristics of the included subjects are summarized in **Supplementary Table S1**. The studies selected in the meta-analysis included cross-sectional, cohort ones. Based on the NOS, all of the studies were moderate or high quality, with an average NOS of 7.06. The details have been shown in **Supplementary Table S2**.

### 3.3 Meta-analysis of cytokines/chemokines

**Figure 2:**
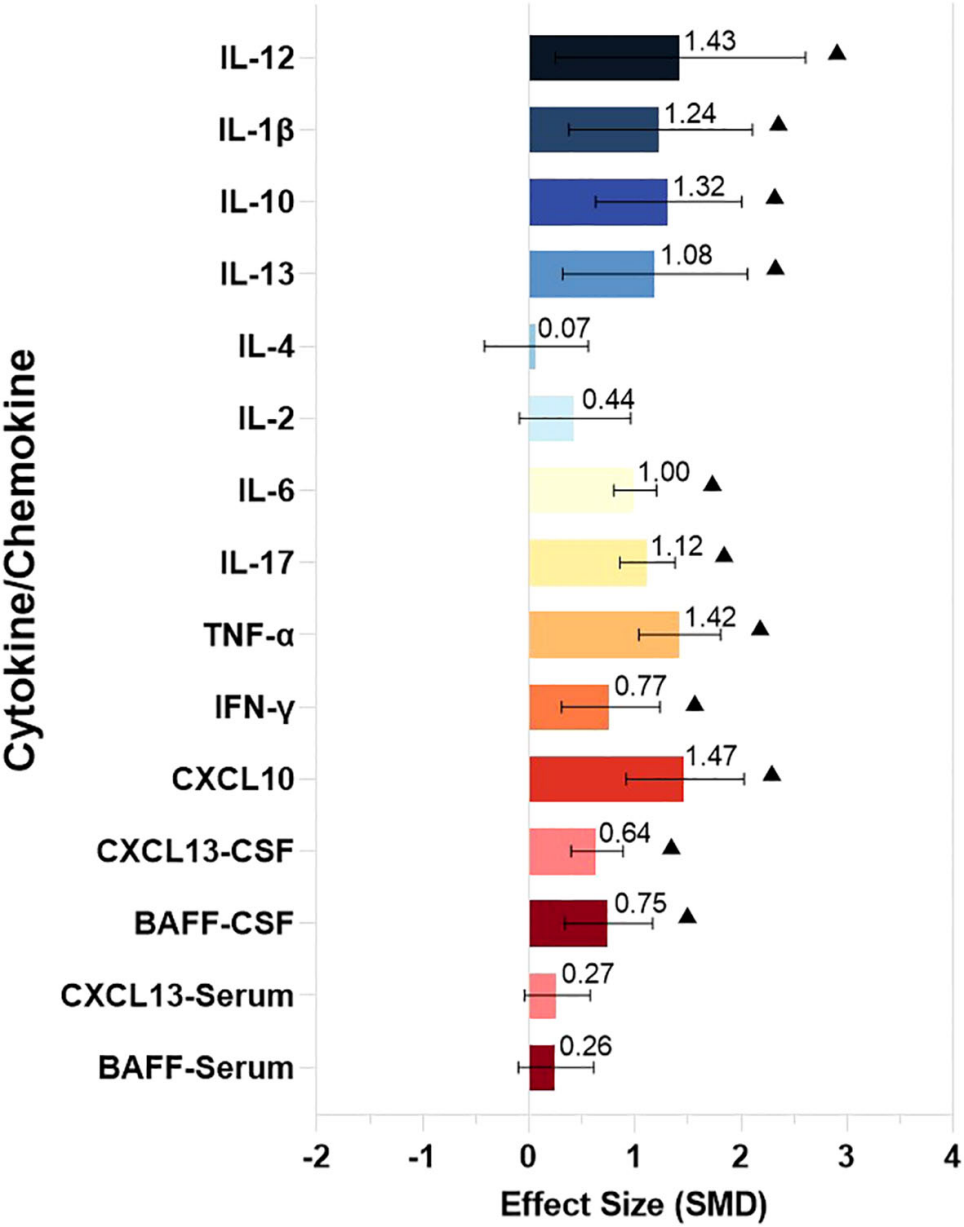
The comparison of CSF and serum levels of the cytokines/chemokines in NMDAR-E. Effect size estimates (SMD) and 95% confidence intervals are represented by colored bars and black error bars, ▲ represents *P* < 0.05. SMD, standardized mean difference; Th, T helper; Treg, T regulatory; BAFF, B cell activating factor; CXCL, C-X-C motif ligand; IFN,interferon; TNF, tumor necrosis factor; IL, interleukin.

We divided the identified cytokines/chemokines into several groups including the B-cell axis, T-cell axis, and broad spectrum based on the distinct roles of associated lymphocytes in the pathogenesis of NMDAR-E. The outcomes of the meta-analysis showed that the levels of 11 different cytokines/chemokines, BAFF, CXCL13, CXCL10, interferon (IFN)-γ, tumor necrosis factor (TNF)-α, IL-17, IL-6, IL-10, IL-13, IL-1β, and IL-12, in the CSF were significantly higher in NMDAR-E patients compared to those in controls. However, levels of IL-2 and IL-4 in the CSF and CXCL13 and BAFF in the serum showed no significant difference. The details are shown in **Table 1** and **Figure 2**.

**Table 1:** Summary of the meta-analysis outcomes for the different cytokines/chemokines.

					Main Effect					Heterogeneity			
Cytokine/chemokine		Sample	Study	N(case/control)	SMD	95%CI	*Z*	*P*		τ²	I² (%)	df	*P*
B cell axis	BAFF	Serum	3	87/50	0.26	-0.10, 0.61	1.42	0.155		<0.01	<0.01	2	0.767
	CXCL13	Serum	4	115/64	0.27	-0.04, 0.58	1.69	0.090		<0.01	<0.01	3	0.447
	BAFF	CSF	3	59/48	0.75	0.34, 1.17	3.59	<0.001		<0.01	<0.01	2	0.929
	CXCL13	CSF	5	259/124	0.64	0.40, 0.89	5.16	<0.001		<0.01	<0.01	4	0.410
Th1 cell axis	CXCL10	CSF	3	29/39	1.47	0.92, 2.03	5.19	<0.001		<0.01	<0.01	2	0.991
	IFN-γ	CSF	5	97/113	0.76	0.22, 1.29	2.74	0.001		0.21	58.91	4	0.119
	TNF-α	CSF	8	159/133	1.42	1.04, 1.81	7.29	<0.001		0.14	48.81	7	0.057
Th17 cell axis	IL-17	CSF	10	214/238	1.12	0.86, 1.37	8.45	<0.001		0.05	30.99	9	0.157
	IL-6	CSF	11	284/278	1.00	0.80, 1.21	9.58	<0.001		0.02	19.42	10	0.259
Th2 cell axis	IL-2	CSF	3	85/90	0.46	-0.15, 1.07	1.48	0.101		0.18	62.97	2	0.133
	IL-4	CSF	3	29/43	0.07	-0.42, 0.56	0.27	0.783		<0.01	<0.01	2	0.611
	IL-13	CSF	3	29/43	1.08	0.15, 2.00	2.27	0.040		0.43	64.65	2	0.031
Treg cell axis	IL-10	CSF	8	188/171	1.32	0.63,2.01	3.74	<0.001		0.83	86.41	7	<0.001
Broad spectrum	IL-1β	CSF	6	138/150	1.25	0.38, 2.11	2.82	0.005		1.02	88.83	5	<0.001
	IL-12	CSF	3	29/43	1.43	0.25, 2.61	2.37	0.018		0.81	74.78	2	0.019

SMD, standardized mean difference; Th, T helper; Treg, T regulatory; BAFF, B cell activating factor; CXCL, C-X-C motif ligand; IFN,interferon; TNF, tumor necrosis factor; IL, interleukin.

Thereafter, for optimal understanding of the results obtained for each cytokine/chemokine, we generalized the key characteristics of our data. Cytokines/chemokines were divided as not statistically significant: the symbol “-” indicated the cytokines/chemokines for which there was no significant evidence (*P* ≥ 0.05) . Three asterisks depicted large effect sizes (SMD ≥0.8); two asterisks, medium effect sizes (0.5 ≤ SMD < 0.8); one asterisk, small effect sizes (0.2 ≤ SMD < 0.5). Significantly higher CSF cytokines/chemokines CXCL10, TNF-α, IL-17, IL-6, IL-10, IL-13, IL-1β, and IL-12 were identified in NMDAR-E patients in comparison with those in control subjects associated with the T-cell axis and mononuclear macrophages, dendritic cells, and other immune cells (**Table 2**).

**Table 2:** Summary of the CSF/serum levels of the cytokines/chemokines in NMDAR-E vs. control subjects.

Group	Cytokine/ chemokine	Sample	Main Source	Main function	Cytokine/chemokine Levels of NMDAR-E vs. control
B cell	BAFF	Serum	Mø, DC,T cells	Regulates B-lymphocyte survival and maturation	–
	CXCL13	Serum	Mø, DC	Selective attraction of B cells	–
	BAFF	CSF	Mø, DC,T cells	Regulates B-lymphocyte survival and maturation	**
	CXCL13	CSF	Mø, DC	Selective attraction of B cells	**
Th1	CXCL10	CSF	Mø,EC,fibroblasts	Chemotaxis of Mø, NK cells, and DC; promotes T cell adhesion to endothelial cells	***
	IFN-γ	CSF	T cells, natural killers cells	Promotes differentiation of Th0 into Th1;stimulates Mø;increases osteoclastogenesis	**
	TNF-α	CSF	Mø, T cells	Increases PMN migration; up-regulates expression of IL-1β,IL-6, and RANKL	***
Th17	IL-17	CSF	Th cells	Stimulates production of TNF-α,IL-6,and IL-1β; increases RANKL expression	***
	IL-6	CSF	Mø,EC,T cells	Increases inflammatory cell migration; increases osteoclastogenesis	***
Th2	IL-2	CSF	T cells	Promotes T cell proliferation and activation; increases NK proliferation	–
	IL-4	CSF	T cells, mast cells	Increases Th0 proliferation; increases IL-10 production; inhibits pro-inflammatory cytokines activity;	–
	IL-13	CSF	Th2 cells	Induces differentiation of monocytes;induces B cell proliferation;Inhibits of inflammation	***
Treg	IL-10	CSF	Mø, Treg	Inhibits Mø antigen-presenting capacity; activates OPG	***
Broad spectrum	IL-1β	CSF	Mø,EC,fibroblasts,DC	Increases inflammatory cell migration; increases osteoclastogenesis	***
	IL-12	CSF	Mø,DC	Promotes differentiation of Th0 into Th1; increases NK proliferation	***

*** - large (SMD ≥ 0.8) and significant (p < 0.05) effect sizes; ** - medium (SMD ≥ 0.5 and SMD < 0.8) and significant (p < 0.05) effect sizes; the symbol “-” was used to identify cytokines/chemokines for which no good evidence could be found;MØ, macrophages ;DC, dendritic cells;EC, epithelial cells;SMD, standardized mean difference; NK, natural killer; PMN, polymorphonuclear; RANKL, receptor activator of nuclear factor-kappa B ligand; OPG, Osteoprotegerin.

#### 3.3.1 Meta-analysis of B-cell axis cytokines/chemokines

BAFF and CXCL13 can recruit and activate B cells, respectively. NMDAR-E CSF BAFF and CXCL13 levels were found to be significantly higher than those of the control with a middle effect size (BAFF: SMD = 0.75, 95% CI = 0.34–1.17, *P* < 0.001; CXCL13: SMD = 0.64, 95% CI = 0.4–0.89, *P* < 0.001). Moreover, the serum BAFF and CXCL13 random-effects model meta-analysis indicated that both did not differ significantly from those of the control (BAFF: SMD = 0.26, 95% CI = -0.10 to +0.61, *P* = 0.155; CXCL13: SMD = 0.27, 95% CI = -0.04 to +0.58, *P* = 0.090) (**Supplementary Figures S1–S4**).

Influence analyses suggested that the effect size for serum CXCL13 was sensitive to the effect of the study reported by Leypoldt et al. (29), and upon omitting this study, the effect size was changed from 0.27 to 0.49, with the 95% CI lower limit changed from -0.04 to 0.08. Thereafter, the case and control groups’ outcomes differed in a significant way (SMD = 0.49, 95% CI = 0.08–0.91, *P* = 0.02). The study by Leypoldt et al. contained a larger sample size than the other three studies and played a key role in a meta-analysis of CXCL13 in the CSF (30) (**Supplementary Figure S2**). The sensitivity analysis of other cytokines and chemokines was robust when omitting either of these articles (**Supplementary Figures S1, S3–S15**).

#### 3.3.2 Meta-analysis of Th1 cell axis cytokines/chemokines

**Figure 3:**
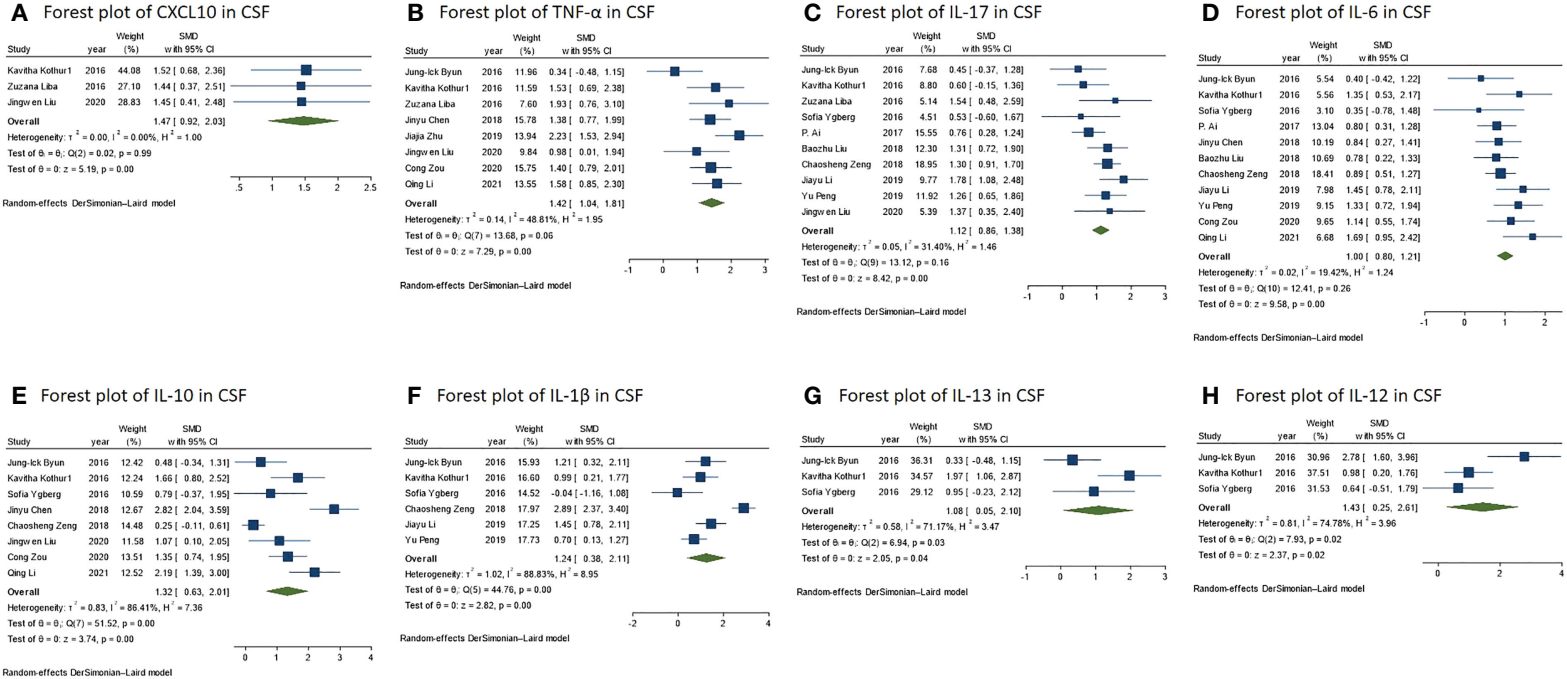
**(A)** Forest plot for the meta-analysis of the CXCL10 level in the CSF of NMDAR-E vs. control. **(B)** Forest plot for the meta-analysis of the TNF-α level in the CSF of NMDAR-E vs. control. **(C)** Forest plot for the meta-analysis of the IL-17 level in the CSF of NMDAR-E vs. control. **(D)** Forest plot for the meta-analysis of the IL-12 level in the CSF of NMDAR-E vs. control. **(E)** Forest plot for the meta-analysis of the IL-10 level in the CSF of NMDAR-E vs. control. **(F)** Forest plot for the meta-analysis of the IL-1β level in the CSF of NMDAR-E vs. control. **(G)** Forest plot for the meta-analysis of the IL-13 level in the CSF of NMDAR-E vs. control. **(H)** Forest plot for the meta-analysis of the IL-12 level in the CSF of NMDAR-E vs. control. CSF, cerebrospinal fluid; NMDAR-E, anti-N-methyl-D-aspartate receptor encephalitis, CXCL, C-X-C motif ligand; TNF, tumor necrosis factor; IL, interleukin.

In comparison to those of the control group, it was discovered that the levels of NMDAR-E Th1 axis-related cytokine TNF-α and the chemokine CXCL10 were significantly higher with a large effect size (**Figures 3A, B**) and IFN-γ with a middle effect size (IFN-γ: SMD = 0.77, 95% CI = 0.31–1.24, *P* = 0.001; TNF-α: SMD = 1.42, 95% CI = 1.04–1.81, *P* < 0.001; CXCL10: SMD = 1.47, 95% CI = 0.92–2.03, *P* < 0.001).

#### 3.3.3 Meta-analysis of Th17 cell axis cytokines/chemokines

**Figure 4:**
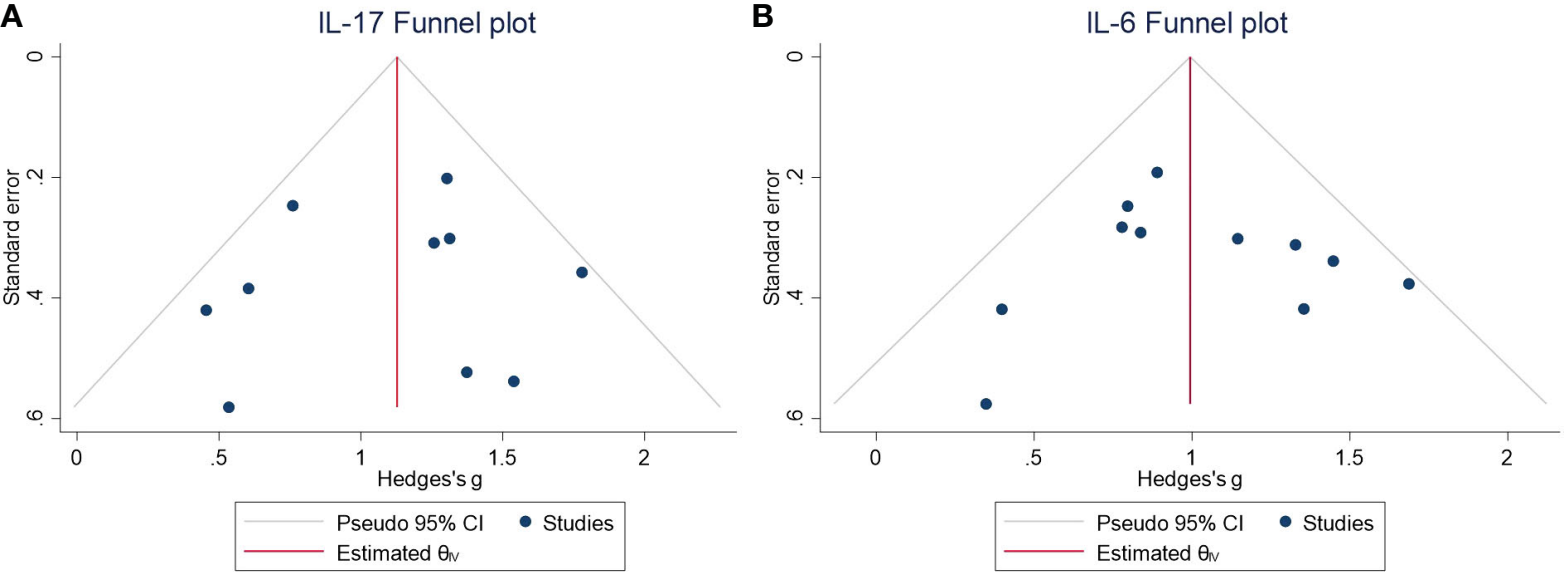
**(A)** Funnel plot for the meta-analysis of the IL-17 level in the CSF of NMDAR-E vs. control. **(B)** Funnel plot for the meta-analysis of the IL-6 level in the CSF of NMDAR-E vs. control. CSF, cerebrospinal fluid; NMDAR-E, anti-N-methyl-D-aspartate receptor encephalitis; IL, interleukin; CI, confidence interval.

The levels of Th17 axis-related cytokines IL-17 and IL-6 were found to be significantly increased (**Figures 3C, D**) (IL-17: SMD = 1.12, 95% CI = 0.86–1.38, *P* < 0.001; IL-6: SMD = 1.00, 95% CI = 0.80–1.21, *P* < 0.001). As there were more than 10 studies related to the cytokines IL-17 and IL-6, we performed publication bias assessment for both IL-6 and IL-17. However, no evidence of publication bias was detected for the meta-analysis of IL-6 (*P* = 0.861, k = 11) and IL-17 (*P* = 0.719, k = 10) by using Egger’s regression and the funnel plots (**Figure 4**).

#### 3.3.4 Meta-analysis of Th2 cell axis cytokines/chemokines

The concentrations of IL-13 associated with the Th2 axis increased with a large effect size, but those of IL-2 and IL-4 did not differ significantly (IL-13: SMD = 1.08, 95% CI = 0.05–2.10, *P* = 0.040; IL-2: SMD = 0.44, 95% CI = -0.09 to 0.96, *P* = 0.101; IL-4: SMD = 0.07, 95% CI = -0.42 to 0.56, *P* = 0.783) (**Figure 3G**). Furthermore, in the IL-13 meta-analysis, included studies were found to be statistically heterogeneous (I^2^ = 71.17%), although the random-effects model was used.

#### 3.3.5 Meta-analysis of Treg cell axis cytokines/chemokines

T regulatory (Treg) cell axis cytokine IL-10 was also found to be significantly higher in comparison to that of the control with a large effect size (**Figure 3E**) (SMD = 1.32, 95% CI = 0.63–2.01, *P* < 0.001). The various included studies were statistically heterogeneous (I^2^ = 86.41%), although the random-effects model was used.

#### 3.3.6 Meta-analysis of the broad-spectrum cytokines/chemokines

We defined IL-1β and IL-12 among the broad-spectrum cytokines, as they are associated with diverse immune cells, and their levels were found to be significantly higher in NMDAR-E compared with those in the control with a large effect size (**Figures 3F, H**) (IL-1β: SMD = 1.24, 95% CI = 0.38–2.11, *P* = 0.005; IL-12: SMD = 1.43, 95% CI = 0.25–2.61, *P* = 0.018). The various included studies were statistically heterogeneous in IL-1β and IL-12 as determined by the random-effects model (IL-1β: I^2^ = 88.83%; IL-12: I^2^ = 74.78%).

## 4 Discussion

In our systematic review and meta-analysis, we found that multiple cytokines and chemokines were shown to be significantly higher in NMDAR-E patients, but we specifically focused on those alterations shown in our meta-analysis due to their higher levels of evidence. We observed that the various cytokines IL-6, TNF-α, IL-10, IL-13, IL-1β, IL-12, and IL-17 and chemokine CXCL10 in the CSF were significantly higher in patients with NMDAR-E, and they were associated with T cells and macrophages and other immune cells (48–50). Interestingly, significant heterogeneity was found in the comparisons of IL-10, IL-1β, IL-12, and IL-13. This heterogeneity could be caused by multiple factors, including age, gender, population, and methodology, as well as the inconsistency of the sample collection time and the healthy state of the control group. However, heterogeneity could not be identified from which research or factor due to the small number of studies included in the meta-analysis. These results reinforced the clinical evidence that NMDAR-E is primarily accompanied by an inflammatory response in the CNS with infiltration of T cells and other immune cells in addition to B cells. In this meta-analysis, we did not notice evidence for the involvement of the cytokines IL-2 and IL-4, but it is possible that if there are more studies related to the cytokines found in NMDAR-E patients and a larger sample size can be used in the future, the results of a new meta-analysis might be different. For the same reason, the meta-analysis excluded several cytokines and chemokines that were reported in less than three studies; however, their exclusion does not imply that they are less clinically relevant. The levels of CD40L, chitinase-3-like protein 1 (CHI3L1), OPN, sCD138, and sCD146 were found to be elevated in the CSF of patients with NMDAR-E and may be associated with severity and prognosis, but more studies to validate their roles are still needed (35, 39, 40, 43, 45).

The meta-analysis included 17 studies published between 2015 and 2021, three of which were published between 2015 and 2016, and included patients only with positive anti-NMDAR antibodies in the serum, which also had clinical features consistent with autoimmune encephalitis. It has been established that patients with NMDAR-E who are negative for antibodies in the CSF may have a delayed onset of antibodies or titers of antibodies could be too low to detect. The delayed onset of anti-NMDAR antibody was previously reported in a 2-year-old child with encephalitis presentation; however, the antibody was always negative in the CSF or serum until 1 year later, and the child developed severe neurological sequelae due to untimely immunotherapy (51). Moreover, based on the findings of other reports, we also found that most patients were probably affected with NMDAR-E, as only antibodies were detected in the serum. They were mostly children and young adults, often with cognitive deficits, catatonia, and speech disturbance. It was found that after the administration of immunotherapy, most of these patients were in remission (52, 53). In addition, some cases only with positive anti-NMDAR antibodies in the serum could also be CSF positive at an early stage due to the time interval between the symptom onset and antibody detection (54). Interestingly, in a prior validation study of a commercial diagnostic kit based on indirect immunofluorescence on transfected cells, a false-positive rate of 1.4% has been reported for the application of serum and cell-based assay (55). When autoantibodies are detected only in the serum, confirmatory tests (e.g., *in vivo* neuronal or tissue immunohistochemistry) should be included (2). However, those people might not require immunotherapy. However, in cases of false-positive antibody tests in the serum and delayed presence in the CSF, it could be necessary to perform a CSF antibody screening and tissue-based assay (2). For patients who are antibody negative but probably suffering from autoimmune encephalitis, novel biomarkers should be explored to aid in proper diagnosis.

B-cell responses have been reported to play a crucial role in the establishment of an NMDAR-E mouse model, according to the different previous studies (11, 19, 56), and its pathogenic mechanism cannot be separated from the involvement of selected cytokines/chemokines. CXCL13, also known as B-lymphocyte chemokine, can effectively recruit B cells into the CNS in NMDAR-E (20, 29, 57). Thereafter, IL-6 together with BAFF and APRIL can exert common and complementary functions in promoting survival, differentiation, and secretion of pathogenic IgG autoantibodies (58, 59). The findings of the meta-analysis indicated that high concentrations of CXCL13, BAFF, and IL-6 in the CSF can effectively lead to the enrichment and activation of B cells in the CNS producing more IgG antibodies, which could explain the reported observations about a higher detection rate of antibodies in the CSF in comparison to that in the serum in NMDAR-E (11, 60). Several selected studies have also shown that high concentrations of CXCL13, BAFF, and APRIL in the CSF were related to poor prognosis (29, 32, 61).

The pathological results in mice showed the infiltration of T cells, B cells, and other immune cells into the CNS (11, 62, 63). In NMDAR-E patients, we found that T cells also play a key role in regulating immune pathogenesis by not only assisting B cells to mature and effectively differentiate into plasma cells secreting specific antibodies resulting in specific neuron lesions associated with the extracellular GluN1 subunit but also forming nonspecific inflammatory responses with other immune cells (10, 13). In addition, the various cytokines/chemokines associated with T lymphocytes that were found to be elevated in our meta-analysis also indicated that T cells can play a key role in NMDAR-E. The different immune cells infiltrating into the CNS can stimulate and constrain each other by secreting diverse cytokines/chemokines, thus forming a complex network of action (64, 65).

It has been found that with the help of Th1 and Th2 axis-related cytokines, CD4+ T cells can differentiate into Th1 and Th2, thus maintaining immune regulation homeostasis (66–68). When the Th1/Th2 cell balance is disrupted, Th1 cells can secrete several pro-inflammatory cytokines to further promote inflammation (69, 70). Interestingly, based on the results of our meta-analysis, effect sizes of Th1-related cytokines were significantly higher than those of cytokines that were Th2 cell related, thus indicating that the Th1/Th2 ratio was imbalanced in NMDAR-E patients. The chemokine CXCL10 can recruit Th1 cells to produce IFN-γ and TNF-α, which can further promote the release of varied cytokines/chemokines and enhance the humoral immune response in the CNS, thus increasing the persistence of the immune response in NMDAR-E (41). IL-17 can damage the integrity of the blood–brain barrier by downregulating the tight junction molecules and promoting astrocytes secreting chemokines that can attract neutrophils to the endothelium (63, 71). Moreover, previous studies have reported that Th17 cells and their secreted IL-17 are pathogenic in multiple sclerosis (MS) patients (48). For NMDAR-E, IL-6 and IL-17 can enhance the differentiation and maturation of Th17 cells and B cells, which can release a variety of cytokines to further enhance the immune response and aggravate the inflammatory response, with IL-17 being associated with poor prognosis (30, 36). In addition, further investigations are needed to evolve novel strategies to target Th17 cells and their associated cytokines and to establish whether they can be used for treatment and improve the prognosis. Treg cells are known for reducing immune responses and tissue damage by encouraging macrophages to secrete IL-10 through the release of IL-13 and for phagocytosing apoptotic cells. The study showed that IL-10 was positively related to the modified Rankin scale (mRS) score in NMDAR-E (41, 72, 73).

In the CNS and peripheral blood of NMDAR-E patients, there are also a large number of immune cells that can generate various cytokines and chemokines. Once the inflammatory response is triggered, a series of immune cells such as endothelial cells, astrocytes, and myeloid cells produce different pro-inflammatory cytokines IL-6, IL-17, and TNF-α, which can synergistically aggravate the inflammatory response (74–76). Furthermore, prior studies in mouse models have revealed that autoimmune antibodies can activate both astrocytes and microglia, and then these cells can release cytokines such as IL-1β and IL-12 to promote the epileptic occurrence and enhance the immune effects of Th1 cells (77, 78). In addition, activated follicular helper T cells and follicular dendritic cells can release CXCL13 to promote the immune response (18). These immune cells can facilitate inflammation, especially in the early stages of autoimmune encephalitis (30, 31).

In conclusion, the stimulation of the central immune response in NMDAR-E is primarily a process of immune disorders mediated by multiple immune cells composed of B cells, Th1 cells, Th17 cells, Treg cells, and related cytokines/chemokines. Th1 cells (related to CXCL10, IFN-g, TNF-a) and Th17 cells (related to IL-17, IL-6), which play key roles in promoting inflammation and promoting B-cell differentiation to plasma cells. Further research should aim to explore the immune pathogenesis process and identify novel immunotherapeutic targets to reduce recurrence and improve prognosis.

## 5 Limitations

This meta-analysis has several limitations. First, as the results of only a small number of studies have been included for each cytokine/chemokine, subgroup meta-analyses could not be conducted, and a meta-regression to explain the high heterogeneity of some of the selected cytokines/chemokines was not carried out. Second, for the meta-analysis of several cytokines/chemokines, only three studies were included, and hence, the results lack robustness. Third, since access to the laboratory data and cytokine results for the individual patient was not available, there may have been several cases with negative CSF anti-NMDA antibodies among the included studies in this meta-analysis. In conclusion, we suggest that multicenter approaches may be necessary to analyze the function of different cytokines/chemokines in the pathogenesis of NMDAR-E.

## Data availability statement

The original contributions presented in the study are included in the article/**Supplementary Material**. Further inquiries can be directed to the corresponding authors.

## Author contributions

YM performed the literature search, analyzed most of the data, and drafted the manuscript. JW analyzed part of the data and wrote the manuscript. SG designed the analyses. ZM analyzed part of the data. YR searched the literature. YX interpreted the results. MW conceived the idea, edited the manuscript, and selected the publication. All authors contributed to the article and approved the submitted version.
