# Supplementary material for: Cytokine/chemokine levels in the CSF and serum of anti-NMDAR encephalitis: A systematic review and meta-analysis
Source: Front Immunol. 2023 Jan 23;13:1064007. doi: 10.3389/fimmu.2022.1064007 (PMC9903132; doi:10.3389/fimmu.2022.1064007)
Supplement: Supplementary file 1 [file DataSheet_1.docx]

**Figure S1:** Meta-analysis and sensitivity analysis of serum BAFF in NMDAR-E vs. controls.


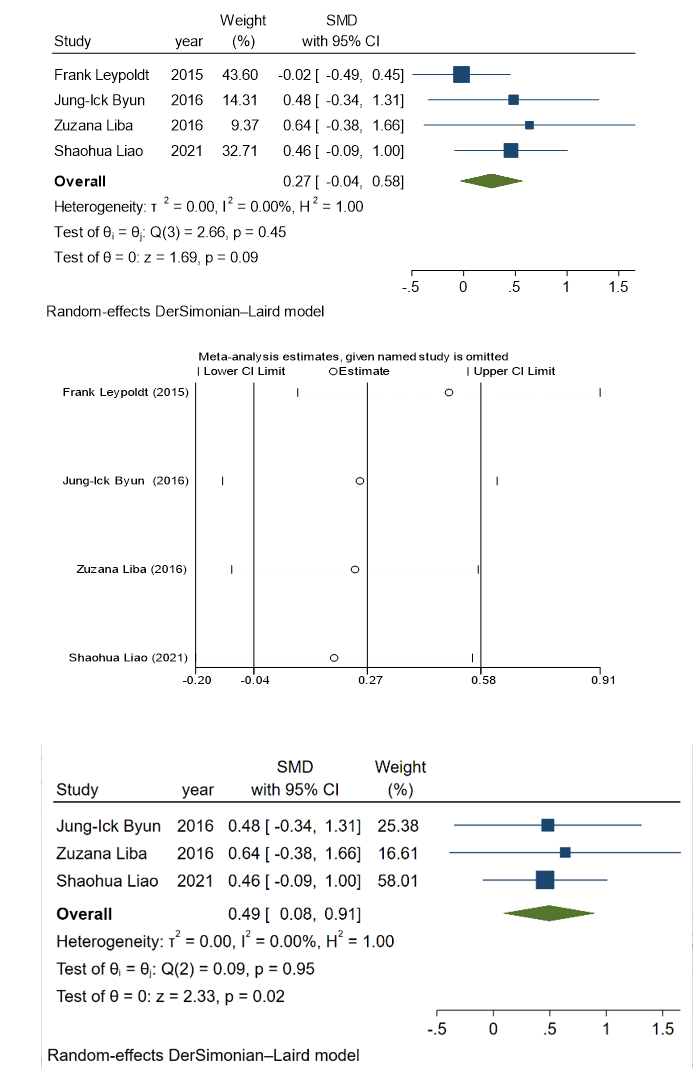


**Figure S2:** Meta-analysis of serum CXCL13 in NMDAR-E vs. controls. The top, middle, and bottom figures show the original meta-analysis of4 observations, sensitivity analysis, and the meta-analysis of 3 observations after removing the study detected as influential study through sensitivity .

**Figure S3:** Meta-analysis and sensitivity analysis of CSF BAFF in NMDAR-E vs. controls.

**Figure S4:** Meta-analysis and sensitivity analysis of CSF CXCL13 in NMDAR-E vs. controls.

**Figure S5:** Meta-analysis and sensitivity analysis of CSF IFN-γ in NMDAR-E vs. controls.

**Figure S6:** Meta-analysis and sensitivity analysis of CSF IL2 in NMDAR-E vs. controls.

**Figure S7:** Meta-analysis and sensitivity analysis of CSF IL4 in NMDAR-E vs. controls.

**Figure S8:** Sensitivity analysis of CSF CXCL10 in NMDAR-E vs. controls.

**Figure S9:** Sensitivity analysis of CSF TNF-α in NMDAR-E vs. controls.

**Figure S10:** Sensitivity analysis of CSF IL-17 in NMDAR-E vs. controls.

**Figure S11:** Sensitivity analysis of CSF IL-6 in NMDAR-E vs. controls.

**Figure S12:** Sensitivity analysis of CSF IL-13 in NMDAR-E vs. controls.

**Figure S13:** Sensitivity analysis of CSF IL-10 in NMDAR-E vs. controls.

**Figure S14:** Sensitivity analysis of CSF IL1βin NMDAR-E vs. controls.

**Figure S15:** Sensitivity analysis of CSF IL-12 in NMDAR-E vs. controls.
