# Supplementary material for: Cytokine/chemokine levels in the CSF and serum of anti-NMDAR encephalitis: A systematic review and meta-analysis
Source: Front Immunol. 2023 Jan 23;13:1064007. doi: 10.3389/fimmu.2022.1064007 (PMC9903132; doi:10.3389/fimmu.2022.1064007)
Supplement: Supplementary file 2 [file DataSheet_2.docx]

**Methods**

We searched databases on June 25, 2022, using the following strategies:

PubMed

| 18 | #8 AND #17 |
| --- | --- |
| 17 | #9 OR #10 OR #11 OR #12 OR #13 OR #14 OR #15 OR #16 |
| 16 | transforming growth factor[Title/Abstract] |
| 15 | tumor necrosis factor-alpha[Title/Abstract] |
| 14 | interferon[Title/Abstract] |
| 13 | interleukin[Title/Abstract] |
| 12 | cytokine[Title/Abstract] |
| 11 | cytokines[MeSH Terms] |
| 10 | chemokine[Title/Abstract] |
| 9 | chemokines[MeSH Terms] |
| 8 | #1 OR #2 OR #3 OR #4 OR #5 OR #6 OR #7 |
| 7 | anti-N-methyl-d-aspartate receptor antibody encephalitis[Title/Abstract] |
| 6 | NMDA receptor encephalitis[Title/Abstract] |
| 5 | N-methyl-d-aspartate antibody encephalitis[Title/Abstract] |
| 4 | Anti NMDAR Encephalitis[Title/Abstract] |
| 3 | anti–N-methyl-d-aspartate receptor encephalitis[Title/Abstract] |
| 2 | Anti NMDA Receptor Encephalitis[Title/Abstract] |
| 1 | Anti-N-Methyl-D-Aspartate Receptor Encephalitis[MeSH Terms] |

Cochrane Library

| #1 | Mesh descriptor: [Anti-N-Methyl-D-Aspartate Receptor Encephalitis] explode all trees |
| --- | --- |
| #2 | (Anti NMDA Receptor Encephalitis):ti,ab,kw |
| #3 | (anti-N-methyl-d-aspartate receptor antibody encephalitis):ti,ab,kw |
| #4 | (Anti NMDAR Encephalitis):ti,ab,kw |
| #5 | (N-methyl-d-aspartate encephalitis):ti,ab,kw |
| #6 | (NMDA receptor encephalitis):ti,ab,kw |
| #7 | #1 OR #2 OR #3 OR #4 OR #5 OR #6 |
| #8 | (cytokine):ti,ab,kw |
| #9 | (interleukin):ti,ab,kw |
| #10 | (interferon):ti,ab,kw |
| #11 | (tumor necrosis factor-alpha):ti,ab,kw |
| #12 | (transforming growth factor):ti,ab,kw |
| #13 | (chemokine):ti,ab,kw |
| #14 | #8 OR #9 OR #10 OR #11 OR #12 OR #13 |
| #15 | #7 AND #14 |

Embase

| #17 | #7 AND #16 |
| --- | --- |
| #16 | #8 OR #9 OR #10 OR #11 OR #12 OR #13 OR #14 OR #15 |
| #15 | ‘transforming growth factor’:ab,ti |
| #14 | ‘tumor necrosis factor-alpha’:ab,ti |
| #13 | interferon:ab,ti |
| #12 | interleukin:ab,ti |
| #11 | cytokine:ab,ti |
| #10 | chemokine:ab,ti |
| #9 | ‘cytokine’/exp |
| #8 | ‘chemokine’/exp |
| #7 | #1 OR #2 OR #3 OR #4 OR #5 OR #6 |
| #6 | ‘nmda receptor encephalitis’:ab,ti |
| #5 | ‘n-methyl-d-aspartate antibody encephalitis’:ab,ti |
| #4 | ‘anti nmdar encephalitis’:ab,ti |
| #3 | ‘anti-n-methyl-d-aspartate receptor encephalitis’:ab,ti |
| #2 | ‘anti-n-methyl-d-aspartate receptor antibody encephalitis’:ab,ti |
| #1 | anti nmda receptor encephalitis’/exp |

Web of Science

TS=(cytokine OR interleukin OR interferon OR Tumor Necrosis Factor-alpha OR Transforming growth factor OR chemokine) AND TS=(Anti NMDA Receptor Encephalitis OR Anti-N-Methyl-D-Aspartate Receptor Encephalitis OR Anti NMDAR Encephalitis OR N-methyl-d-aspartate antibody encephalitis OR NMDA receptor encephalitis OR anti-N-methyl-d-aspartate receptor antibody encephalitis)
