# Supplementary material for: Cytokine/chemokine levels in the CSF and serum of anti-NMDAR encephalitis: A systematic review and meta-analysis
Source: Front Immunol. 2023 Jan 23;13:1064007. doi: 10.3389/fimmu.2022.1064007 (PMC9903132; doi:10.3389/fimmu.2022.1064007)
Supplement: Supplementary file 3 [file Table_1.docx]

**Table S1:** Characteristics of studies included in the meta-analysis of CSF and serum cytokines and chemokines in patients with NMDAR-E compared to control subjects

| **Study** | **Country** | **Case subjects** | | | | **Control subjects** | | | | **Cytokine(s) included in the meta-analysis** | **Method for cytokine measurement** |
| --- | --- | --- | --- | --- | --- | --- | --- | --- | --- | --- | --- |
|  |  | Condition | N | Mean /Medium  age (years) | Male (%) | Condition | N | Mean /Medium  age (years) | Male  (%) |  |  |
| Leypoldt et al. 2015 | Spain | NMDAR-E | 167 | - | - | noninflammatory neurological disorders | 25 | - | - | CXCL13 | ELISA |
| Byun et al. 2016 | South Korea | NMDAR-E | 14 | 30 | 28.6 | noninflammatory neurological disorders | 10 | 48 | 40 | IL1b, IL-2, IL-4, IL-6, IL-10, IL-12, IL-13, IL-17, IFN-γ, TNF-α, and CXCL13 | ELISA  And Bio-Plex Cytokine Assay system |
| Kothur et al. 2016 | Australia | NMDAR-E | 11 | 6 | 54.5 | noninflammatory neurological disorders | 20 | 4.9 | 55 | IFN-γ,TNF-α, CXCL10,IL-2,IL-4, IL-13,IL-6,IL-17, BAFF,CXCL13, IL-10, IL-12(p70),IL-1b | multiplexed fluorescent bead-based immunoassay detection (MILLIPLEX) |
| Liba et al. 2016 | Czech | NMDAR-E | 9 | 13 | 11.1 | noninflammatory neurological disorders | 10 | 12 | 20 | IFN-γ,TNF-α, CXCL10,,IL-17, BAFF,CXCL13, | Luminex multiple bead technology(Luminex-100 system)  And ELISA |
| Ygberg et al. 2016 | Sweden | NMDAR-E | 4 | 5.9 | 25 | Non-encephalitis | 13 | 1.1 | 46.2 | IL-1b,IL-4, IL-6, IL-10, IL-12p70, IL-13,IL-17, IFN-γ | Bio-Plex Pro, Life Science Bio-Rad, |
| Deng et al. 2017 | China | NMDAR-E | 40 | 28 | - | noninflammatory neurological disorders | 20 | 34.3 | - | BAFF | ELISA |
| Ai et al. 2017 | China | NMDAR-E | 33 | 35.52 | 45.5 | noninflammatory neurological disorders | 38 | 39.32 | 44.7 | IL-6,IL-17, | unclear |
| Chen et al. 2018 | China | NMDAR-E | 33 | 34.8 | 42.4 | noninflammatory neurological disorders | 21 | 34.81 | 38.1 | IL-6,IL-10,TNF-α | ELISA |
| Liu et al. 2018 | China | NMDAR-E | 24 | 36.42 | 58.3 | noninflammatory neurological disorders | 31 | 37.42 | 48.4 | IL-6,IL-17 | unclear |
| Zeng et al. 2018 | China | NMDAR-E | 60 | 33.5 | - | noninflammatory neurological disorders | 60 | 37.3 | - | IL-2，IL-6，IL-10，IFN-γ，IL-1b，IL-17，CXCL13 | Bio-Plex Cytokine Assay System and ELISA |
| Li et al. 2019 | China | NMDAR-E | 24 | 34.02 | 41.67 | noninflammatory neurological disorders | 21 | 36.48 | 52.4 | IL-6，IL-17，IL-1b | ELISA |
| Peng et al. 2019 | China | NMDAR-E | 25 | 35.5 | 44 | noninflammatory neurological disorders | 26 | 38.8 | 50 | IL-6，IL-17，IL-1b | ELISA |
| Zhu et al. 2019 | China | NMDAR-E | 27 | 34.67 | 44.44 | noninflammatory neurological disorders | 25 | 37.45 | 48 | TNF-α | ELISA |
| Liu et al. 2020 | China | NMDAR-E | 10 | 27 | - | noninflammatory neurological disorders | 9 | 38 | - | IL-17,IL-10, CXCL10,TNF-α | MILLIPLEX MAP multiple biomarker detection technology |
| Zou et al. 2020 | China | NMDAR-E | 33 | 34.8 | 42.4 | noninflammatory neurological disorders | 21 | 35.2 | 38.1 | IL-6,IL-10,TNFα | ELISA |
| Liet et al. 2020 | China | NMDAR-E | 23 | 29.6 | 34.78 | noninflammatory neurological disorders | 17 | 27.5 | 47.06 | IL-6,IL-10,TNFα | ELISA |
| Liao et al. 2021 | China | NMDAR-E | 39 | - | - | Health control | 42 | - | - | CXCL10,CXCL13,IL-10,BAFF | premixed multiplex system human magnetic luminex assays. |
