# Supplementary material for: Cytokine/chemokine levels in the CSF and serum of anti-NMDAR encephalitis: A systematic review and meta-analysis
Source: Front Immunol. 2023 Jan 23;13:1064007. doi: 10.3389/fimmu.2022.1064007 (PMC9903132; doi:10.3389/fimmu.2022.1064007)
Supplement: Supplementary file 4 [file Table_2.docx]

**Supplementary Table S2:** Quality of studies included in meta-analysis assessed using Newcastle-Ottawa Scale.

| **Study** | **Design** | **Selection** | **Comparability** | **Outcome** | **Newcastle-**  **Ottawa Scale**  **Score** |
| --- | --- | --- | --- | --- | --- |
| Leypoldt et al. 2015 | cross-sectional | *** | * | ** | 6 |
| Byun et al. 2016 | cross-sectional | **** | * | ** | 7 |
| Kothur et al. 2016 | cross-sectional | **** | * | ** | 7 |
| Liba et al. 2016 | cohort | *** | ** | ** | 7 |
| Ygberg et al. 2016 | cross-sectional | ** | * | ** | 5 |
| Deng et al. 2017 | cross-sectional | *** | * | ** | 6 |
| Ai et al. 2017 | cross-sectional | *** | * | *** | 7 |
| Chen et al. 2018 | cross-sectional | *** | * | ** | 6 |
| Liu et al. 2018 | cross-sectional | **** | * | ** | 7 |
| Zeng et al. 2018 | cross-sectional | **** | * | *** | 8 |
| Li et al. 2019 | cross-sectional | **** | ** | ** | 8 |
| Peng et al. 2019 | cross-sectional | **** | * | *** | 8 |
| Zhu et al. 2019 | cross-sectional | **** | * | ** | 7 |
| Liu et al. 2020 | cross-sectional | **** | * | *** | 8 |
| Zou et al. 2020 | cross-sectional | **** | ** | ** | 8 |
| Liet al. 2020 | cross-sectional | **** | ** | *** | 9 |
| Liao et al. 2021 | cross-sectional | *** | * | ** | 6 |
